# Using the Red List of Ecosystems and the Nature-based Solutions Global Standard as an integrated process for climate change adaptation in the Andean high mountains

**DOI:** 10.1098/rstb.2022.0326

**Published:** 2024-06-10

**Authors:** L. Vasseur, A. Andrade

**Affiliations:** ^1^ UNESCO Chair on Community Sustainability: from Local to Global, Department of Biological Sciences, Brock University, 1812 Sir Isaac Brock Way, St Catharines, ON, Canada L2S 3A1; ^2^ Conservation International-Colombia, Carrera 13 no. 71–41, Bogotá, Colombia 110221; ^3^ Commission on Ecosystem Management, International Union for the Conservation of Nature, 28 Rue Mauverney, 1196 Gland, Switzerland

**Keywords:** nature, climate change, adaptation, resilience, sustainability

## Abstract

Under anthropogenic pressures and climate change, most ecosystems are showing signs of reduced resilience. Unfortunately, some are more at risk of collapse and, without interventions, they may lose biodiversity, ecological integrity and ecosystem services. Here, we describe two tools that were developed under the auspices of the International Union for Conservation of Nature, the Red List of Ecosystems and the Nature-based Solutions Global Standard, and their capacity to first identify the ecosystems at risk of collapse in a nation and then develop solutions based on nature to improve their resilience. Nature-based solutions include, for example ecosystem-based adaptation, where solutions are developed to meet the needs of the local people while protecting nature to ensure greater resilience of the social–ecological system, not only the natural ecosystem. We discuss through a case study in the Andean high mountains and páramo social–ecological system how these approaches have been used in Colombia. We then discuss lessons learned and challenges that may reduce the capacity of a community to initiate such interventions, such as national policies and funding restrictions. We also discuss through another early case in Ecuador the importance to adapt these types of interventions to the geographical and cultural context of the social–ecological systems.

This article is part of the theme issue ‘Bringing nature into decision-making’.

## 1. Introduction

The current dual crisis of biodiversity loss and climate change is affecting all ecosystems across the world, as well as human well-being, as the United Nations' Intergovernmental Platform on Biodiversity and Ecosystem Services (IPBES) stated in its Global Ecosystem Assessment report in 2019 [1]. The resilience of social–ecological systems (SESs) is a major concern as they may no longer be able to recover their functions after a disturbance. The concept of resilience is not new in many disciplines, such as engineering, ecology, psychology, physics and social sciences. In his classic paper, Holling [2] introduced this concept to ecological systems, which has been since appropriated by social researchers and for interdisciplinary applications (e.g. social–ecological resilience, [3]). The concept of resilience has also become part of climate change and natural disasters as well as biodiversity conservation research (e.g. [4]). Across the world, bodies ranging from communities to national governments have developed different adaptation and response strategies to cope with the perturbations. The aim for societies is to maintain functional and productive SESs to ensure their sustainability. For many communities, coping has become a priority by implementing pragmatic short-term solutions [5]. Berkes & Jolly [6] associate ‘coping’ with short-term, often reactive, strategies and activities, while ‘adaptation’ entails strategies over the longer term. These authors stress that using coping strategies over the long-term can actually be maladaptive in the context of climate change. Many short-term adaptation strategies, which are in fact coping strategies, do not consider the SES but rather the social components, neglecting to assess the adaptive capacity of the ecological system to sustain changes, thus leading to its possible collapse [7,8].

Most SESs around the world are now showing some degree of loss of resilience [9]. It then becomes a question of identifying which ones are close to collapse owing to different causes such as land-use change, invasive species, pollution or climate change, among others [1]. Such an SES may become a priority, but may not be able to reverse to its original state and therefore may be transformed into a novel system. In recent years, in the climate change sphere, for example, the terms adaptation and transformative adaptation have been increasingly used as some SESs need to change or adapt to ensure the sustainability of societies that rely on them [10]. It may be necessary to move away from gradual adaptation in response to changes to transformative adaptation, with incremental impacts of the perturbations, especially under the current climate change scenarios. Here, we define transformative adaptation as ‘changes that fundamentally alter the entire system's ecological and/or social properties and functions' [10, p. 116]. In the case of collapse, societies may have no choice to migrate to survive or, if a novel SES evolves, societies may be able to change to sustain their livelihoods [10].

Since the adoption of the Ecosystem Approach by the United Nations (UN) Convention on Biological Diversity [11], especially, during the last 12 years, the International Union for Conservation of Nature (IUCN), through the work of its expert scientists from academia and other organizations, has developed and standardized two major tools. The first one, the Red List of Ecosystems (RLE), adopted in 2014, contributes to assessing and monitoring ecosystem status. The second one, adopted in 2020 is called Nature-based Solutions (NbS) and aims to address multiple social–ecological challenges in an integrated manner. Both are considered as policy standards that contribute to the implementation of robust and sustainable interventions.

The RLE is a global framework for monitoring risks to ecosystems. It assesses whether ecosystems have reached an extreme stage of degradation (a state of collapse), using the following criteria: (A) reductions in geographical distribution; (B) restricted ecosystem distribution; (C) rates of environmental degradation (of natural and anthropogenic nature); (D) rates of decline in biotic processes and species interactions; and (E) quantitative estimates of ecosystem collapse risk [12]. The RLE offers a quantitative means of assessing the risk of ecosystem collapse and identifying threatened ecosystems, by measuring ecosystem transformation, degradation, and functional change [13]. Through the RLE, it is possible to identify the most eftective management pathways to reduce risks and loss of biodiversity. Assessments can be undertaken at regional, national or subnational level.

Currently, over 4000 ecosystem types have been assessed in 63 countries [14]. The RLE has been adopted by the Global Biodiversity Framework as headline indicator of Goal A [15]. A second and related advance of the RLE is the development of the Global Ecosystem Typology, which is a hierarchical classification system for all terrestrial, aquatic and marine ecosystems [16]. It has been also recognized as a reference classification for the System of Environmental Economic Accounting (SEEA) Ecosystem Accounting (and adopted by India and Brazil, for example; https://seea.un.org/news/ncaves-project-countries-progress-piloting-ecosystem-accounting-and-valuation-ecosystem).

The concept of NbS has become popular to answer to multiple social–ecological crises such as land degradation, biodiversity loss and climate change. The term NbS is defined by the IUCN as ‘actions that aim at addressing significant societal challenges by sustainably managing, restoring or protecting nature, while benefiting both human well-being and biodiversity’ [17, p. xii]. In March 2022, the United Nations Environment Assembly (UNEA) of the United Nations Environment Programme adopted the concept of NbS with a slight modification, ‘actions to protect, conserve, restore, sustainably use and manage natural or modified terrestrial, freshwater, coastal and marine ecosystems which address social, economic and environmental challenges effectively and adaptively, while simultaneously providing human well-being, ecosystem services, resilience and biodiversity benefits' [18, p. 2]. The NbS Global Standard was adopted by the IUCN in 2020. It is framed on the Ecosystem Approach [11]. It is a facilitative tool aiming at helping design and implement robust and sustainable interventions, contributing to human well-being and sustainability while conserving the ecological system. The standard assesses the extent to which a proposed intervention qualifies as an NbS, by using eight criteria and related 25 indicators. These criteria consider the need to assess the targeted societal challenge(s), the required spatial considerations, the involvement of stakeholders and governance, the particularities of navigating and balancing trade-offs, the promotion of adaptive management, the enhancing of positive outcomes for biodiversity, society and economy, and mainstreaming within national policy and sectors [19].

The IUCN RLE and the Global Standard for NbS have been developed at different times and separately. However, their synergistic application can contribute by first prioritizing ecosystems that are most at risk and then, with involvement from local communities, co-develop NbS interventions that can enhance resilience of SESs. This unique approach has yet to be fully integrated.

In this opinion piece, we first further describe the RLE and NbS concepts, process, and the Global Standard and how they can support transformative adaptation and change when they are integrated. Then, this process is examined through a National RLE Assessment [20] and a case study in high mountain ecosystems and páramos in Colombia. These ecosystems have been identified as highly vulnerable under the RLE to climate change impacts, owing to land-use change and unsustainable land-use practices. Changes in temperature and rainfall patterns are affecting the well-being of local populations and the provision of water for human consumption. The steps to implement NbS are described in relation to transformation, and the criteria of the NbS Global Standard have been assessed [21]. This particular case study holds significant relevance for countries like Colombia that possess extensive Andean landscapes, as a substantial portion of the population relies on the vital ecosystem services provided by high mountain ecosystems and páramos.

Finally, we discuss some issues important to consider in NbS, such as trade-offs, to sustain transformative adaptation of SESs. We discuss another early case study, in Ecuador, also located in high mountain ecosystems, where a similar approach can be applied, but in a different social, political, ecological and cultural context, underlying the need to define the interventions at the right scale and context for sustainable solutions.

## 2. Ecosystems beyond resilience

At the core of the IUCN Red List of Ecosystems (RLE) lie the categories and criteria. As previously mentioned, the five criteria are used to assess the current state of an ecosystem. These criteria serve as benchmarks for categorizing ecosystems based on their susceptibility to risk. Two of these criteria centre on spatial indicators of ecosystem collapse: Criterion A evaluates declining geographical distribution, while Criterion B focuses on restricted geographical distribution. The remaining two criteria delve into functional indications of ecosystem collapse: Criterion C scrutinizes environmental degradation, while Criterion D assesses the disruption of biotic processes and interactions. Furthermore, by integrating multiple threats and symptomatic cues, a model of ecosystem dynamics can generate quantitative estimations of the likelihood of collapse (Criterion E) [22]. This quantitative risk assessment then allows determination of which of the categories an ecosystem should be classified. The eight risk categories encompass a spectrum of states: Collapsed (CO), Critically Endangered (CR), Endangered (EN), Vulnerable (VU), Near Threatened (NT), Least Concern (LC), Data Deficient (DD) and Not Evaluated (NE).

Criterion E (quantitative analysis that estimates the probability of ecosystem collapse within 50 years) of the RLE quantifies the risk as a probability of collapse for a period of 100 years (with 50% being labelled as Critical, 20% as Endangered, and 10% as Vulnerable) [12]. For example, using the IUCN RLE assessment in British Colombia, Canada, DellaSala *et al*. [23] report that the core inland temperate rainforest primary forest may collapse within 9 to 18 years owing to intensive logging activities (that have doubled since the 1970s). The collapse of these ecosystems would have severe consequences on some endangered species such as woodland caribou in Canada. Lake Burullus in the Nile Delta in Egypt has also been assessed using the RLE criteria. This wetland ecosystem has been listed under the Ramsar Site Convention since 1988, as well as being a protected area, and is considered an Important Bird Area. Since then, however, and despite its protection, the lake has been impacted owing to agricultural water discharge with massive loads of nutrients, which has accelerated the eutrophication of this shallow lake owing in part to the change in water salinity [24]. The RLE assessment indicates that owing to the construction of a highway along its north shore, combined with agricultural and urban effluents, the status of Lake Burullus should be considered Endangered, and without urgent actions to reduce human pressures from urban and agricultural activities, the ecosystem may collapse. The identification of ecosystems at risk of collapse, using the RLE, therefore can help policy-makers and planners identify and implement NbS to build resilience. NbS, in such cases, can help improve biodiversity, ecosystem integrity, and ecosystem services through restoration and effective adaptive ecosystem management.

The risk assessment of terrestrial ecosystems in Colombia indicates that 25% of them are at critical risk levels (Category CR), which significantly affects biodiversity and the ecosystem services they provide, as well as the well-being of the communities depending on them. Among these ecosystems with high levels of risk, more than 18 million hectares have vanished, to the extent that only 3% of the country's untransformed ecosystems currently remains [25,26].

Identifying ecosystems with high risk of degradation or collapse is a first step, but what can be expected when the system is at the point of collapse? When biological diversity and ecosystem functions decline to a certain threshold, the system becomes vulnerable to any additional disturbance [27]. Many ecosystems are now threatened not only because of human activities and disturbances but also owing to climate change, which exacerbates these other pressures and provokes system collapse. Under such conditions, the ecosystem loses its resilience and can become non-functional. Either the ecosystem naturally evolves into a novel ecosystem or it loses all its functions and services, leading to socio-economic tensions and potentially collapse of the communities that rely on this ecosystem. Under a novel ecosystem, the communities may have to change their livelihoods, which can bring new pressure on the ecosystem. If the ecosystem has completely collapsed, there may be a need for direct human action to attempt the re-establishment of some of the functions and services that previously existed. However, this may not always be possible, especially if disturbances that led to the collapse persist [27]. Transforming an ecosystem to improve its functions and services can be quite daunting. One of the best ways to recover or transform an ecosystem is by NbS. In the next section, a case study in a high altitude ecosystem describes the process of assessment of the status of an ecosystem using the RLE and how NbS are currently used to re-establish the health of the ecosystem.

## 3. Nature-based solutions and prioritizing most-threatened landscapes

As explained, NbS are any actions that address challenges that societies are facing by protecting, sustainably managing and restoring natural or modified ecosystems to improve resilience. These challenges can stem from environmental and/or climate changes and usually threaten the sustainability of the SESs, not only the ecosystems. Therefore, NbS aim to simultaneously provide human well-being and biodiversity benefits. It is important to underline the intricate link between NbS and the Ecosystem Approach [11]. Indeed, NbS principles have been framed based on the twelve principles of the Ecosystem Approach, and integrate issues from community needs and importance of governance to adaptive management of ecosystems for biodiversity and ecosystem services [28]. In fact, while the Ecosystem Approach did not refer to SESs, the principles are directly in line with the SES concept. NbS target seven societal challenges that are currently affecting all nations, including climate change adaptation and mitigation, disaster risk reduction, reversing ecosystem degradation and biodiversity loss, human health, socio-economic development, food security and water security [19]. Many of these challenges are interrelated and need to be considered together [29].

While communities may be highly interested in developing interventions that integrate NbS, it is mainly at the governmental level that some of the first steps can begin, to initially determine which societal challenges and ecosystems are to be prioritized. NbS usually consider the large landscape approach, which can contain different ecosystems, some of which may be near collapse while others are degraded managed lands to be restored. Understanding these components at the landscape level requires assessments of the whole SES and the threats for each of its components. This is where RLE, as previously presented, represents one of the most effective tools that can be used to define the ecosystems at greatest risk. From there, through community engagement, it is possible to integrate the societal challenges, thus moving to an SES approach.

The connection between the two standards is through Criterion 3 of the IUCN Global Standard for Nature-based Solutions, which requires that interventions must result in a net gain in terms of biodiversity and ecosystem integrity. Consequently, the foundational assessment for any intervention should encompass, at a minimum, an evaluation of external threats to ecosystems and their susceptibility to degradation and collapse. This assessment is integral to establishing the baseline. Furthermore, this criterion ensures that interventions not only enhance ecological integrity but also contribute to an elevation in species diversity [19].

## 4. A case study: Colombian high mountain SESs in transformation

In Colombia, all 81 identified terrestrial ecosystem types have been assessed using the RLE standard. Among them, 25% of continental ecosystems (representing 20 ecosystem types) are listed as at high risk and 17% (14 ecosystem types) as endangered**.** They represent nearly half of Colombian ecosystems, with conditions that threaten their integrity and therefore their ability to provide ecosystem services [25,26]. The reduction of natural ecosystem extent and integrity is mainly due to the expansion of the agricultural frontier, but additional threats arise from climate change, which are becoming more and more relevant in Andean Colombia. Among these services, water provision and regulation take precedence. Decline in water availability and rainfall patterns during the year and temperature increase in altitudes above 3000 m a.s.l. are major concerns, leading to great risk of ecosystem collapse. The human communities who rely on these ecosystem services are therefore threatened as much as the ecosystems in which they live.

High mountain ecosystems and páramos have been considered as priorities for conservation and sustainable management (Target 15.4 of the Sustainable Development Goals [30]). In Colombia, located over 2740 m a.s.l., these ecosystems compose 3.6% of the total surface area of the country, but they represent an important biodiversity hotspot called the tropical Andes [31]. The main ecosystem types present in the Colombian Andes are: páramos and subpáramos, cloud forests, Andean forests, sub-Andean forests, semiarid enclaves and mountain wetlands. páramos represent only 2.6% of the territory, but provide significant ecosystem services, mainly water and water regulation as well as a carbon sink [32]. Based on the RLE assessment, páramos are categorized as Critically Endangered, while all other ecosystems are classified as Vulnerable [25,26]. Climate change and land-use changes are affecting the ecological integrity of these fragile ecosystems, especially the páramos. For example, over 15% of the páramos have been transformed into agricultural land (pastures, crops, and tree plantations), while 45% of the territory is under the national System of Protected Areas [33]. Human water consumption and hydropower further impact these ecosystems, adding pressure on the city of Bogotá and its 21 surrounding municipalities, including the farmers. With over 10 million people living in this territory, these ecosystems are critical for the socio-economic development of the country.

Increasing average annual temperatures and less predictable rainfall patterns, along with land-use transformations, are affecting the integrity of those ecosystems and increasing the risk of collapse [34]. The decline in the capacity of ecosystems to provide services, as well as habitat for key species, in high mountain ecosystems and moorlands (páramos) further threatens their resilience [33]. The high vulnerability of high mountain ecosystems to climate change impacts has led the Colombian government to develop policies and plans to not only reduce the impacts from climate change but also adapt and restore these ecosystems to improve resilience (e.g. National Climate Change Policy, Integrated National Adaptation Plan, National Restoration Plan, Development Plan of the Department of Cundinamarca). For example, the Integrated National Adaptation Pilot was implemented between 2006 and 2011 to support the government of Colombia in its efforts to define and implement pilot adaptation measures and policy options to anticipate climate change impacts [35]. This project was followed by another one (2015–2021) in the high mountain areas of Chingaza, Sumapaz and Guerrero, which provide water to Bogotá and the surrounding municipalities, with the objective of improving water regulation in the higher part of the watersheds to ensure sustainable water provision to the city of Bogotá [36].

The second project used NbS for adaptation as the basic concept to deal with the societal challenges of the communities living in the high mountain areas, which included improvement of sustainable agriculture and efficient use of water. All interventions carried out in the field were orchestrated by local communities and stakeholders who had vested interests owing to the current societal challenges, especially maintaining their livelihoods. Knowledge dissemination among communities residing in this territory severely impacted by climate change was an important step to engage people in co-defining solutions. This step was completed through farming adaptation techniques such as agroecology and greenhouse vegetable production with more effective water regulation, as well as production of free-range chickens and honey to improve the livelihoods of the local people. At the same time, to further reduce the impacts of water regulation, biodiversity and rural production, ecological restoration was initiated through the recovery of areas of ecological importance for connectivity, water regulation, protection of native vegetation, control and eradication of invasive species, and addition of mammal shelters and multifunction strips. At the farm level, the construction of water retention ponds and the introduction of irrigation systems helped reduce water wastage. Some of the priority actions were also directed towards reversing the páramo degradation. Shifting from cattle ranching and potato monocultures to more diversified and adaptive agricultural practices, such as regenerative agriculture and agroforestry, helped further reduce the impacts on this fragile SES.

Diversifying livelihoods and improving food security, with sustainable, low-water and climate-resilient uses (e.g. eco-tourism, payment for ecosystem services, fodder banks as dry season feed reserves) significantly helped reduce poverty of the local people. By stabilizing their income throughout the year and thus the household economy a sense of well-being within local communities was restored [36]. The adaptation pilot projects carried out in this SES have been recognized as examples of ecosystem-based adaptation actions that have gone beyond adaptation to provide employment opportunities and increased food supply at farm level [37]. Updating land-use plans to integrate adaptation with NbS also contributed to protect water provision, based on the identification and assessment of climate risks and the recognition of the ‘*Ecological Adaptive Territorial Structure*’ as a geographical network of spaces that support essential ecological processes at the landscape level, based on the existing ecosystem types maps [38]. The enhancement of spatial planning, indicating areas that need to shift current land uses towards conservation, restoration and modification from livestock and potato cultivation practices to more sustainable forms of production, was a relevant outcome of the project. The data were necessary to guide adaptation beyond mere biodiversity conservation and towards the maintenance of ecosystem structure and functioning. These land-use plans are currently strategically adopted by 22 municipalities for water provision and regulation. Under the leadership of several of the beneficiaries and local authorities for these interventions, planning and actions are maintained. The reduction of the human pressures on the natural ecosystems, including the provision of water, has improved the livelihoods of the local people, with more sustainable and environmentally friendly techniques resulting in an improvement of the water provision for the lower lands, including the city of Bogotá. The ultimate objective is to maintain ecological integrity and ecosystem health in the long term, including all relevant structural elements of the landscape, to ensure the conservation and recovery of ecosystem services in páramo and the high mountains, which are highly vulnerable to climate change, including issues related to water cycle regulation, water quantity and quality, groundwater recharge, risks of natural hazards and erosion.

The NbS for adaptation process used in this case study are also innovative in that they introduce climate information systems and participatory monitoring of key indicators such as ecological and social conditions. Risk assessment in this case includes climate risk based on climate change scenarios as the main input to determine the vulnerability of the SESs and their adaptive capacity. The community is engaged in the priority actions seeing benefits not only in the short term but also in the long term. The project has been developed at multiple scales, including users upstream and downstream and establishing local forums among multiple water users.

The update of the municipal plans regarding land use to include adaptation and introducing climate risk management is also helping reduce the risks in the other communities relying on the high mountain ecosystems for water use and even food production [36]. It is important to note that the project was selected as a case study by the Commission on Ecosystem Management of IUCN for an analysis of the Global NbS Standard self-assessment tool and showed near compliance to all the indicators.

The process described here in Colombia requires to be tested and applied in other localities to assess the possibility for extending such approaches to other areas. This is currently being examined in the high mountains and the páramos of the Chimborazo in Ecuador, where a similar scenario is at play. Local people have been locked into potato monoculture and the use of sheep and cattle pastures, which are currently degrading the páramo ecosystem and the entire SES. These Quichua communities live in a low-income generation economy where climate change is now threatening their SES owing to alterations in decline in annual precipitation, change in rainfall pattern and the melting of the Chimborazo. Increased erosion and decline in soil fertility add to the threats of their current livelihoods. Some limited activities have been initiated such as replacing the sheep by the dwarf alpaca and planting native trees in cattle pastures, but they remain small owing to lack of knowledge and limited funding (activities have been supported by a tripartite collaboration between three universities: Brock University in Canada, Fujian Agriculture and Forestry University in China and the Escuela Superior Politecnica de Chimborazo in Ecuador) as well as governmental unwillingness to act. The importance of governmental support and potential changes in policies to allow for transformative changes is essential. A major difference that may be at play in Ecuador is the fact that the capital, Quito, is far from the Chimborazo Mountain and páramos and so this region's influence on policies remains weak. Therefore the successful application of RLE and NbS Standards can in part demonstrate the power of the approach as well as the potential to influence regional and national governments and their policies. In the case of Colombia, the national government is already thinking about scaling up this case study, again demonstrating the usefulness and effectiveness of this approach to enhance resilience of these SESs.

## 5. Moving forward in nature-based solutions for transformative changes (lessons learned and challenges)

In this paper, we have discussed how the IUCN RLE Standard can help identify ecosystems that are endangered or at great risk of collapse and the capacity through the IUCN Global NbS Standard to reduce the risk of an ecosystem to collapse. These two approaches should be integrated as Criterion 3 of the NbS Global Standard requires the assessment of the SES. The vulnerability assessment of ecosystems through the lens of RLE helps understand which ones may be most at risk, and therefore influence future actions, planning and policies [22,25,26]. Recent work has demonstrated that climate change can be integrated into the RLE assessment, although this may not be possible everywhere, owing to limited climate data [39]. The ability to address the various types of risks and drivers that can bring an ecosystem to collapse presents a great advantage to support nations in developing integrated planning that considers both biodiversity and climate change at the same time, as recently underlined in the Kunming–Montreal Global Biodiversity Framework [15]. Indeed, the RLE is included as the first headline indicator for Goal A of the Global Biodiversity Framework. Assessing the connection between biodiversity and climate change as a dual crisis allows a better understanding of the SES complexity and reduces the danger for fragmentated actions. Oversimplifying vulnerability assessments can lead to fragmented responses and maladaptation. SESs are hypercomplex, and indicators used to assess vulnerability are often oversimplified, assume linear relationships among variables and remain static while systems are dynamic [40]. For ecosystems that are near to collapse, mainly owing to climate change, such as the high tropical Andes, there is a need for strengthening an interdisciplinary research agenda with strong policy formulation and cross-sectorial cooperation including direct engagement of the communities that have to deal with the consequences of these risks [41,42].

Similarly, to effectively use NbS as a transformative adaptation process, complexity and uncertainty of the SES facing collapse due to various drivers including climate change require going beyond the simple analysis of climatic variables, to understand the connections with other ecosystem components and services provided, including the socio-economic and cultural aspects of the communities that rely on these ecosystems. The criteria of the NbS Global Standard also underline the importance of inclusive governance (Criterion 5) and therefore the involvement of the communities throughout the intervention cycle and its monitoring [19]. Seddon *et al.* [43] argue that NbS can support socio-economic exposure and sensitivity through restoration and protection of natural ecosystems as well as transforming agroecosystems into agroforestry. Involving communities in such transformations, as shown in our case study, can help improve livelihoods. However, a risk remains as to ensure that local land rights and cultural practices are respected as well as the equity of the various groups that compose the communities [43]. To ensure equity and fairness, all NbS including adaptation strategies should have adequate socio-ecological research and knowledge generation based on transdisciplinary knowledge and knowledge co-creation. Cooperation and collaboration among all stakeholders are essential to reduce conflicts and understand trade-offs that may need to be made considering their needs and those of the ecosystems to maintain service provision [43].

The NbS for adaptation case study in Colombia followed a comprehensive approach encompassing key stages for design, implementation and evaluation. This involved the assessment of climate change impacts, integration of a socio-ecological vulnerability assessment, identification of appropriate adaptation measures with the communities, design, implementation and evaluation, as well as monitoring. This process is outlined in Donatti *et al*. [44]. The assessment of the intervention′s transformative potential was also conducted using fundamental indicators for measuring transformative changes within SESs [10]. These indicators encompassed path shifting, restructuring, considerations across multiple scales, innovation, system-wide integration and persistence [10]. The outcomes of this assessment indicated that the high mountain ecosystems intervention in Colombia yielded significant multiscale adaptation benefits, with other indicators falling within the medium to high range. These aforementioned indicators have been effectively applied to various case studies in the Pacific, as demonstrated by [45]. This application has contributed to the enhancement of the IUCN Global NbS Standard, further solidifying the relevance and effectiveness of these indicators in evaluating and standardizing NbS worldwide.

The advantage of NbS is their capacity to move from biodiversity conservation to climate change adaptation as well as contribute to sustainable development goals. By considering the needs of the local people, goals like ending poverty and hunger can be fulfilled. To do so, future climate change policies must focus on identifying and discussing trade-offs to balance communities' needs and conservation issues, as well as the interests of the different stakeholders. NbS allow the appreciation and respect of the multiple values of nature and culture. NbS recognize the importance of high-quality interventions by identifying synergies ands trade-offs. Transformative changes using NbS may also require different financing and valuation, beyond market-based approaches. Since NbS can provide a wider range of solutions and delivery of ecosystem services, it can ‘provide low-cost solutions to many climate change related impacts and offer key advantages over engineered solutions' [43, p. 7]. However, it does not preclude the use of engineering solutions or grey infrastructure in some cases depending on the context. Cost effectiveness may play a major role in defining which solutions may be best or combined to improve sustainability. To be effective, NbS should be financially supported, especially for marginalized and developing nations. In the case of Colombia, support of the government and funds from donors allowed effective actions. Barriers can still remain at the national and political levels where policies are anchored in a system that does not allow transformative changes. Institutional norms can also become a barrier to changing regulations and policies to allow NbS to be effective. While NbS may have become a new fad for governments and is now included in the Kunming–Montreal Global Biodiversity Framework, embarking on this process requires them to be open to major changes to allow transformative changes to occur.

## 6. Conclusion: avoiding ecosystem collapse through nature-based solutions and transformative changes

In this paper, we examine how RLE and NbS Standards can create an avenue for countries to develop long-term and sustainable solutions that may reduce the possible collapse of ecosystems at risk, as well as improving the well-being of local communities. With the increasing need to deal with multiple crises and societal challenges, business as usual is not an option for many ecosystems. Our case study demonstrates the importance of first understanding the state of the social ecological system, the anthropogenic drivers as well as the climate-related variables to design solutions that can be sustainable and socially acceptable to local people while positively impacting the surrounding ecosystems, in this case the city of Bogotá. Can the same process be used in other locations? The potential of using in Ecuador an approach similar to the one in Colombia can help assess the more global capacity of these Standards to help more SESs that are in risk of collapse, mainly due to climate change. The contextual differences between Colombia and Ecuador are of great interest and underline the importance for climate change adaptation to be enacted at the local level. It also demonstrates that NbS remain context-dependent and further research needs to be conducted to better understand the limitations that can be faced by communities to ensure the survival of their SESs.

## Data accessibility

This article has no additional data.

## Declaration of AI use

We have not used AI-assisted technologies in creating this article.
